# Quality comparison of electronic versus paper death certificates in France, 2010

**DOI:** 10.1186/1478-7954-12-3

**Published:** 2014-02-17

**Authors:** Delphine Lefeuvre, Gérard Pavillon, Albertine Aouba, Agathe Lamarche-Vadel, Anne Fouillet, Eric Jougla, Grégoire Rey

**Affiliations:** 1 INSERM, CépiDc, Le Kremlin-Bicêtre, France; 2 AP-HP, Paris, France; 3 Institut de veille sanitaire, Saint Maurice, France

**Keywords:** Death certificate, Causes of death, Electronic certification, Quality

## Abstract

**Background:**

Electronic death certification was established in France in 2007. A methodology based on intrinsic characteristics of death certificates was designed to compare the quality of electronic versus paper death certificates.

**Methods:**

All death certificates from the 2010 French mortality database were included. Three specific quality indicators were considered: *(i)* amount of information, measured by the number of causes of death coded on the death certificate; *(ii)* intrinsic consistency, explored by application of the International Classification of Disease (ICD) General Principle, using an international automatic coding system (Iris); *(iii)* imprecision, measured by proportion of death certificates where the selected underlying cause of death was imprecise. Multivariate models were considered: a truncated Poisson model for indicator (i) and binomial models for indicators (ii) and (iii). Adjustment variables were age, gender, and cause, place, and region of death.

**Results:**

533,977death certificates were analyzed. After adjustment, electronic death certificates contained 19% [17%-20%] more codes than paper death certificates for people deceased under 65 years, and 12% [11%-13%] more codes for people deceased over 65 years. Regarding deceased under and over 65 respectively, the ICD General Principle could be applied 2% [0%-4%] and 6% [5%-7%] more to electronic than to paper death certificates. The proportion of imprecise death certificates was 51% [46%-56%] lower for electronic than for paper death certificates.

**Conclusion:**

The method proposed to evaluate the quality of death certificates is easily reproducible in countries using an automatic coding system. According to our criteria, electronic death certificates are better completed than paper death certificates. The transition to electronic death certificates is positive in many aspects and should be promoted.

## Introduction

Causes of death statistics are essential data to monitor population health, undertake epidemiological studies, and international comparisons. High-quality mortality data are needed in this respect, and the European Commission has expressed the importance of producing recommendations on methods that improve the quality and international comparability of cause of death statistics [1].

**Figure 1:**
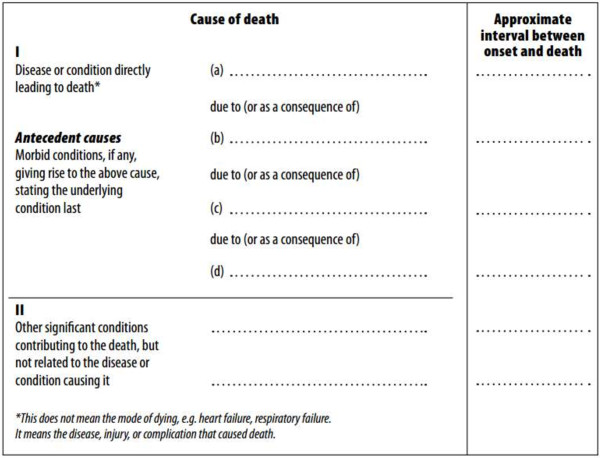
**International form of medical certificates of cause of death.** French death certificates, in compliance with the World Health Organization (WHO) international standards, are composed of two parts: Part I is dedicated to the reporting of diseases related to the train of events leading directly to death, and Part II is dedicated to the reporting of contributory conditions not directly involved in the death process.

French death certificates, in compliance with the World Health Organization (WHO) international standards, are composed of two parts: Part I is dedicated to the reporting of diseases related to the train of events leading directly to death, and Part II is dedicated to the reporting of contributory conditions not directly involved in the death process (Figure 1).

Causes of death data are centralized at the French Epidemiological Center for the Medical Causes of Death (CépiDc - Inserm). The death certificates are coded automatically by the international software Iris (Additional file 1: Iris software) in order to select the underlying cause of death (UCD); complex cases are reviewed by nosologists. The UCD is defined as “the disease or injury which initiated the train of morbid events leading directly to death, or the circumstances of the accident or violence which produced the fatal injury.” One single underlying cause is selected for each death, following the General Principle and rules described on the International Statistical Classification of Diseases (ICD) and Related Health Problems, 10^th^ revision [2] (Additional file 2: Rules for mortality coding). The poor quality and comparability of medical cause of death data are mainly due to the lack of training of certifiers. The death certificate, the underlying cause concept, and the rules that are applied to determine it, are all defined by WHO, thus following an international standard that ensures quality and comparability. The development of electronic certification has several aims: *(1)* to facilitate the physician’s certification process with online explanations (description of each part of the death certificate and illustrative examples of the correct way to fulfill them), *(2)* to limit errors when filling in the death certificate and hence improve data reliability, *(3)* to provide a much quicker process to health surveillance and alert systems, *(4)* to strengthen data security and confidentiality, and *(5)* to reduce costs [3]. Moreover, in order to facilitate the use of electronic certification, a learning mode allows physicians to practice before writing a real death certificate.

In 2007, electronic certification was introduced in France, with the objective, among others, of increasing the quality of the causes of death certification process [4]. Currently, around 5% of deaths certificates are electronically certified [3]. The system is run on a complete voluntary basis and is, at present, primarily dedicated to hospitals and health institutions rather than ambulatory medicine. The consent is sought from institutions and not from physicians. The French electronic certification process uses a Web application that only requires an Internet connection and secured physician identification parameters [5].

The aim of this study was to compare the quality of electronic certification versus classical paper certification. In this respect, it proposes a reproducible methodology to assess the level of quality based on the analysis of intrinsic characteristics of death certificates. These types of studies are essential at present, as many countries are planning to implement electronic death certification and automated coding systems [6].

To assess the quality of information contained in death certificates, two main approaches exist. The first consists of comparing the selected UCD with a gold standard; this approach is called “content validity” [7]. The gold standard may be based on data information such as an autopsy report or a clinical evaluation during the last hospitalization [8,9]. Other investigations have been based on samples of cases histories used to complete and code death certificates. The resulting UCD is then compared with a reference coding [10-13]. Such approaches are complex, costly, and difficult to reproduce as a routine quality-checking process. The other approach is called “criterion validity.” It does not compare data to a gold standard but consists of evaluating intrinsic characteristics of death certificates, looking for errors leading to inconsistencies [14,15]. Very few studies have used the criterion validity approach. In France, this type of evaluation has never been conducted. At the international level, Mathers et al. compared data quality between countries according to the completeness of reporting and the proportion of deaths attributed to ill-defined diseases and attributed to each country a high, medium, or low quality level [16]. As it does not measure the accuracy of the reported causes of death, this method was considered as a very partial evaluation of the quality of cause of death certification by other experts [17]. Consequently, in our study, we developed a specific reproducible and global method.

## Methods

### Data sources

The present study is a population-based study on routine death certificates.

All electronic and paper death certificates received and coded by the CépiDc during year 2010 were taken into account, provided that at least one cause was mentioned. Neonatal death certificates regarding children deceased before 28 days of age were excluded because of their specificity. For each death, available data were birth date, death date, gender, place of occurrence, and region of death, type of certificate (electronic or paper), and all the causes of death reported by the physician certifier, coded with the tenth revision of the ICD (ICD-10).

All the included certificates were coded with Iris software. Five automated coding systems (ACS) have been developed throughout the world. The United States was the first country to develop an ACS. Sweden, France, and Hungary followed. All of these systems are compatible with the US system. They code the causes of death and select the underlying cause of death according to the ICD-10 rules and guidelines. However, these systems are dependent on the language used for the causes of death reporting. This is why Iris was developed: it provides a system both compatible with the US system and usable in any language. Iris is now used by several countries such as Sweden, France, Germany, Canada, South Africa, Israel, and Luxembourg. The CépiDc is one of the four institutions that participate in the Iris collaborative project. For each certificate, Iris documents the rules used to select the underlying cause of death through the Automated Classification of Medical Entities (ACME) system (Additional file 1: Iris Software, [18,19]). For few death certificates (that are too complex or have iatrogenic problems), Iris cannot select the UCD automatically. However, this concerns less than 1% of death certificates. The UCD for these certificates is chosen by nosologists, and they were not included in analyses.

### Quality assessment

Three indicators were used to measure certification quality: amount of information contained in the death certificates, intrinsic consistency and imprecision level (Table 1). Death certificates were analyzed for each of these indicators.

**Table 1:** Indicators retained to evaluate intrinsic quality of death certificates

**Type of indicator**	**Assessing methodology**	**Example**
Information quantity		
Average number of conditions	Mean of codes written on death certificate	
Intrinsic consistency		
Correct completion	Application of General Principle	Part I:
		a) Sepsis
		b) **Pneumonia**
Several sequences	Application of Rule 1	Part I:
		a) Respiratory arrest
		b) **Lung cancer**, heart failure
No logical sequence	Application of Rule 2	Part I:
		a) **Cerebrovascular accident**
		b) Alzheimer’s disease
Imprecision		
Underlying cause of death imprecise	Imprecise underlying cause of death, marked by following ill-defined codes: R00-R99 (except R95), I469, I99, I959, J960, J969.*	Part I:
		a) Multiple organ failure
		b) **Cardiac arrest**

* I469 = Cardiac arrest, unspecified, I959 = Hypotension, unspecified, I99 = Other and unspecified disorders of circulatory system, J960 = Acute respiratory failure, J969 = Respiratory failure, unspecified, R00-R99 = Symptoms, signs and abnormal clinical and laboratory findings, not elsewhere classified, R95 = Sudden infant death syndrome.

Causes in bold are selected in each case.

The amount of information was defined as the number of ICD-10 codes reported on the death certificates [2]. Even if information quantity is not, in itself, a measure of certification quality, this indicator is a marker, all other characteristics being equal, of the physician’s attention in completing the death certificate.

The intrinsic consistency was evaluated by the proportion of death certificates where the General Principle applied. When the General Principle did (respectively, did not) apply, death certificates were considered as consistent (respectively, inconsistent). When the General Principle did not apply, two cases were distinguished according to the selection rules, applying Rule 1 when more than one causal sequence was reported in Part I of the death certificate, and Rule 2 when no causal sequence was correctly ordered (often because the certifier filled the death certificate from the top down) (Additional file 2: Rules for mortality coding) [2]. As there is no consensus about the quality level of death certificates revealed by the application of one of these two rules, their distribution was studied only for a descriptive purpose.

Imprecision was measured by the proportion of death certificates in which the UCD selected by ICD rules was ill-defined. An ill-defined UCD was identified as one of the following ICD-10 codes: R00-R99 (symptoms, signs, and abnormal clinical and laboratory findings, not elsewhere classified), except R95 (sudden infant death syndrome); I469 (cardiac arrest, unspecified), I99 (other and unspecified disorders of circulatory system), I959 (hypotension, unspecified), J960 (acute respiratory failure), or J969 (respiratory failure, unspecified).

### Statistical analyses

Paper death certificates and electronic death certificates were compared according to each criterion by the way of univariate and multivariate analysis designed to control the effect of several variables. As the death certificates analyzed contained at least one code and because number of causes by death certificate was a counting variable, zero-truncated Poisson models were fitted to model count data for which the value zero cannot occur [20]. Intrinsic consistency and imprecision level were investigated using a log-linear binomial model estimating relative risks (RR) comparing electronic versus paper death certificates [21]. Both models included the type of certificate (electronic versus paper) and socio-demographic and death-related characteristics: age (< 65 years versus ≥ 65 years), gender, place of occurrence of death (hospital or private clinic versus home and other places), and region of death (22 regions and all overseas regions merged) as explanatory variables. Models for the amount of information and intrinsic consistency were also adjusted on UCD in six classes corresponding to ICD chapters: neoplasms, cardiovascular diseases, respiratory diseases, gastrointestinal diseases, violent deaths, and other diseases. A stepwise variables selection procedure was performed in order to determine the final model. As multiple pathologies frequently affect old people and because one unique underlying cause of death is harder to determine in this population [22], interaction was tested between type of death certificate and age. Given its statistical significance, age-specific type of death certificate effects were estimated in both models. As a sensitivity analysis, an adjustment on age in 10-year classes was performed, but the results were unchanged. Analyses were performed with SAS® software 9.3.

## Results

**Figure 2:**
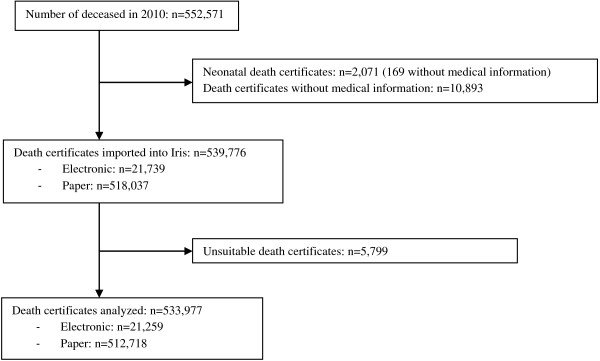
**Inclusion process for death certificates.** Among the 552,571 deceased in 2010, CépiDc received and coded 541,678 death certificates, of which 1,902 were neonatal death certificates. 539,776 death certificates were included and processed by Iris, and 533,977 were analyzed for quality assessment, including 21,259 electronic death certificates and 512,718 paper death certificates.

Among the 552,571 deceased in year 2010, CépiDc received and coded 541,678 death certificates, of which 1,902 were neonatal death certificates (Figure 2). 539,776 death certificates were included and processed by Iris; 533,977 could be analyzed for quality assessment, including 21,259 electronic death certificates and 512,718 paper death certificates.

### Information quantity

Out of the 533,977 death certificates analyzed, the average number of codes on electronic death certificates (3.99) was 20% [19%-21%] higher than on paper death certificates (3.39) (Table 2). After adjustment, electronic death certificates recorded 19% [17%-20%] more codes than paper certificates in people deceased under 65 years and 12% [11%-13%] more codes than paper certificates in people deceased over 65 years.

**Table 2:** Number of causes by certificate (results from zero-truncated Poisson models)

	**Mean (standard deviation)**	**Univariate analysis**	**Multivariate analysis**	
			**All-ages type effect**^**1**^	**Age-specific type effect**^**2**^
		**RN [IC **_**95%**_**]**	**RN [IC **_**95%**_**]**	**RN [IC **_**95%**_**]**
All	3.41 (1.89)			
Type				
Electronic, all ages	3.99 (2.13)	1.20 [1.19-1.21]	1.14 [1.13-1.15]	
Paper, all ages	3.39 (1.88)	1.00	1.00	
Electronic, <65 years	3.86 (1.96)	1.25 [1.23-1.27]		1.19 [1.17-1.20]
Paper, <65 years	3.17 (1.76)	1.00		1.00
Electronic, ≥65 years	4.04 (2.19)	1.19 [1.18-1.20]		1.12 [1.11-1.13]
Paper, ≥ 65 years	3.44 (1.91)	1.00		1.00
Place of death				
Home or elsewhere	3.07 (1.78)	0.81 [0.81-0.82]	0.82 [0.82-0.82]	0.82 [0.82-0.82]
Hospital or private clinic	3.67 (1.94)	1.00	1.00	1.00
Age				
<65 years	3.20 (1.77)	0.91 [0.91-0.92]	0.89 [0.89-0.89]	0.89 [0.89-0.89]
≥65 years	3.47 (1.92)	1.00	1.00	1.00
Underlying cause of death				
Violent deaths	3.58 (2.05)	1.07 [1.06-1.07]	1.16 [1.15-1.17]	1.16 [1.15-1.17]
Cardiovascular diseases	3.46 (1.89)	1.02 [1.02-1.03]	1.05 [1.04-1.05]	1.05 [1.04-1.05]
Respiratory diseases	3.58 (1.85)	1.07 [1.06-1.07]	1.06 [1.05-1.06]	1.06 [1.05-1.06]
Gastrointestinal diseases	3.89 (1.98)	1.17 [1.16-1.18]	1.16 [1.15-1.16]	1.16 [1.15-1.16]
Others	3.23 (1.99)	0.95 [0.94-0.95]	0.99 [0.99-1.00]	0.99 [0.99-1.00]
Neoplasms	3.39 (1.75)	1.00	1.00	1.00

RN: Relative number of causes by certificate.

^1^Model adjusted on age, type of certificate, place of occurrence of death, gender, initial cause of death, and region of death (data not shown).

^2^Same model as ^1^ with age-specific type effect.

### Intrinsic consistency

343,214 (64.3%) of all death certificates analyzed contained a unique morbid sequence applying the General Principle (Table 3). Without adjustment, the General Principle could be applied 5% [4%-6%] more to electronic than to paper death certificates. After adjustment, the General Principle could be applied more frequently to electronic than to paper death certificates. Regarding the deceased under age 65, the General Principle could be applied 2% [0%-4%] more frequently to electronic than to paper death certificates. As for the deceased over 65 years, the difference rose to 6% [5%-7%]. Results were similar when considering only death certificates with a precise UCD.

**Table 3:** Distribution of the application of the General Principle among death certificates (results from log-binomial models)

	**General principle**	**Univariate analysis**	**Multivariate analysis**	
			**All-ages type effect**^**1**^	**Age-specific type effect**^**2**^
	**N(%)**	**RR [CI **_**95%**_**]**	**RR [CI **_**95%**_**]**	**RR [CI **_**95%**_**]**
All	343214 (64.28)			
Type				
Electronic, all ages	14345 (67.48)	1.05 [1.04-1.06]	1.05 [1.04-1.06]	
Paper, all ages	328869 (64.14)	1.00	1.00	
Electronic, <65 years	3531 (63.64)	1.09 [1.07-1.11]		1.02 [1.00-1.04]
Paper, <65 years	60270 (59.23)	1.00		1.00
Electronic, ≥65 years	10814 (68.89)	1.06 [1.05-1.07]		1.06 [1.05-1.07]
Paper, ≥65 years	268599 (65.36)	1.00		1.00
Place of death				
Home or elsewhere	145196 (63.35)	0.98 [0.97-0.98]	0.97 [0.97-0.98]	0.97 [0.97-0.98]
Hospital or private clinic	198018 (64.97)	1.00	1.00	1.00
Age				
Mean (std)	77.7 (15.5)			
<65 years	63801 (59.46)	0.91 [0.90-0.91]	1.02 [1.01-1.02]	1.02 [1.01-1.02]
≥65 years	279413 (65.49)	1.00	1.00	1.00
Underlying cause of death				
Violent deaths	12916 (34.96)	0.62 [0.62-0.63]	0.63 [0.62-0.64]	0.63 [0.62-0.64]
Cardiovascular diseases	103182 (71.75)	1.28 [1.27-1.29]	1.29 [1.29-1.30]	1.29 [1.29-1.30]
Respiratory diseases	26105 (78.07)	1.39 [1.38-1.40]	1.40 [1.39-1.41]	1.40 [1.39-1.41]
Gastrointestinal diseases	17091 (74.46)	1.33 [1.32-1.34]	1.33 [1.32-1.34]	1.33 [1.32-1.34]
Others	94855 (68.83)	1.23 [1.22-1.24]	1.24 [1.23-1.25]	1.24 [1.23-1.25]
Neoplasms	89065 (56.01)	1.00	1.00	1.00

Relative risks and 95% confidence interval.

^1^Model adjusted on age, type of certificate, place of occurrence of death, gender, initial cause of death, and region of death (data not shown).

^2^Same model as ^1^ with age-specific type effect.

Among the 190,763 death certificates in which the General Principle did not apply, 104,597 (54.8%) needed application of Rule 1. Electronic death certificates required Rule 1 in 62.4% of cases, whereas the corresponding proportion for paper death certificates was 52.6%. After adjustment, Rule 1 applied more often to electronic than to paper death certificates: RR = 1.20 [1.17-1.25] and 1.03 [1.00-1.05] for people deceased under and over 65 years, respectively.

### Imprecision

There were 1.8% electronic death certificates (384/21,259) for which the UCD chosen by Iris was imprecise, compared to 6.4% for paper death certificates (32,628/512,718). The crude difference was significant: RR = 0.28 [0.26-0.31]. After adjustment, results were confirmed, without any significant interaction between age and type of certificate: overall, the risk of an imprecise UCD was 51% (RR = 0.49 [0.44-0.54]) lower for electronic than for paper death certificates.

## Discussion

This study showed that electronic death certificates were better completed in terms of data quality than paper death certificates. Indeed, the General Principle was applied more frequently to electronic than to paper death certificates. In addition, underlying causes of death derived from electronic death certificates were less often imprecise. Furthermore, electronic death certificates contained more information than paper death certificates.

Information quantity cannot be considered, in itself, as a measure of certification quality. Nevertheless, the number of conditions coded is meaningful: it partly reflects the willingness of the physicians to fill in death certificates. It suggests that the physicians generally give more importance to an electronic document than a paper document, possibly because they suppose that the information will be used for epidemiological purposes. From the data analyst point of view, additional information provides perspective for multiple cause analyses, which tend to be developed for complex diseases such as diabetes [23,24].

As the General Principle is more often applied on electronic death certificates, we can assume that the online explanations help physicians to better complete these certificates. It is also possible that the electronic certification, as it is only accessible through a secure connection, prevents the certifier, who should be a physician, from delegating this task to non-physicians (e.g., medical students who have not been taught to complete death certificates). It certainly increases the confidentiality of the causes of death declaration process and possibly increases the overall certification quality.

When the General Principle is not applied, Rule 1 appears more frequently on electronic than on paper death certificates. On the one hand, this finding confirms that morbid sequences are more often logically ordered on electronic certificates: Rule 1 is applied if several logical sequences are declared on part 1 of the death certificates. The latter result could reflect the higher number of codes on these certificates because Rule 1 application requires more than two codes. However, this result is also observed when considering only death certificates containing three codes or more. On the other hand, Rule 2 deals both with cases of top-down sequence or very inconsistent death certificates. These two cases refer to different levels of inconsistency that were not distinguished by the automatic method. Thus, strictly ordering the importance of the two selection rules that both indicate inconsistency of death certificates would be questionable.

In the ICD-10, other rules are defined that we did not take into account. Following selection Rule 3, if the condition selected by the General Principle or by Rule 1 or Rule 2 is obviously a direct consequence of another reported condition, this primary condition must be selected. As we considered that application of the General Principle defined intrinsic consistency, Rule 3 did not prevent a death certificate from being considered as correctly completed. Other rules described in ICD-10 are modification rules that enable improvement of the usefulness and precision of mortality data. They are applied after selection rules. In fact, we considered the application of these rules indirectly when studying imprecision. Imprecise UCD show that despite application of modification rules, UCD remains imprecise.

An adjustment on major UCD categories was made to study the amount of information and intrinsic consistency of death certificates. Some diseases like violent deaths, a frequent cause of death among young people, seem harder to correctly certify following WHO guidelines by physicians. The General Principle applied less often to the death certificates for which the UCD was violent death: the UCD has an impact on quality. However, a death certificate completed improperly could also influence UCD. Thus, including the UCD variable in our models could lead to overadjustment. As a sensitivity analysis, we adjusted the models without including UCD as explanatory variables, and the associations between indicators and type of death certificates were unchanged.

Intrinsic consistency and imprecision were studied by Lu [14] and Myers [15], who also worked only on conditions listed on death certificates. However, except for their definition of major error, which corresponds to the application of Rule 1, the measures proposed by these authors, such as identifying the mechanism written on death certificates, cannot be routinely done easily.

Since the aim was to compare quality data of electronic versus paper death certificates and to use the established method for future evaluations, we needed a simple and reproducible method. Indicators were chosen for their ease of use and their potential impact on mortality statistics. This automated method allowed work on a wide sample. Indeed, we were able to assess the quality of data over a whole year, which is more statistically powerful, and allowed us to adjust the results according to many factors. Moreover, it would have been very difficult to build a representative sample of mortality in France, including clinical data over the whole territory. This method could be used to compare quality of data between countries using the same coding system (automated selection program). In France, electronic certification will regularly be evaluated during its development. This method could also potentially be used to evaluate more precisely the geographical and temporal distribution of the quality of death certificates or the effect of an educational intervention for medical students [15,25,26].

The main limitation of the method used is that it did not take into account the whole coding process but only the choice of the UCD. Indeed, the preliminary coding phase is the attribution of an ICD code to a medical expression. The quality differential corresponding to this phase between electronic and paper certification was not evaluated. However, it is to be expected that the translation from a text to an ICD code is easier when no interpretation of the certifier’s handwriting has to be done.

Furthermore, this method is available when access to electronic or paper death certificates exists, which usually happens only in developed countries.

The study results are possibly affected by a confounding bias, because the medical establishments that adopt electronic certification earlier than others are likely to be more comfortable with technology and more interested in the purpose of certification. Therefore, a better-quality certification could be more attributable to the certifier than to the way of certification.

More specifically, quality of death certification could be associated with the type of institution (teaching hospital, local hospital, or private clinic). Unfortunately, it is impossible to stratify or adjust the model on the type of institution, as this variable is not recorded for paper certificates. However, over the study period, electronic death certification was used by all types of institutions, which suggest that the bias, if it exists, should not be strong.

This study is the first comparing data quality of electronic versus paper death certificates using an automated and reproducible method. Henceforward, the expertise of nosologists who deal with both kinds of certificates might allow us to enhance accuracy criteria, continuing under the constraint of a simple evaluation method. This type of study could be completed by qualitative research on the knowledge, attitudes, practices, and preferences of certifying physicians in relation to electronic versus paper death certification. The findings of such research would be useful in designing broader interventions to improve the implementation of electronic death certification in France as well as in international settings.

## Conclusion

To conclude, in addition to the shortening of the certification process, electronic certification was revealed as an improvement over paper certification in terms of quantity and quality of data. Electronic certification should be developed as widely as possible and international recommendations should encourage electronic certification in order to develop it throughout the world, thus increasing both alert capacities and data quality.

## Abbreviations

ACME: Automated Classification of Medical Entities; ACS: Automated coding system; ICD: International Classification of Disease; UCD: Underlying cause of death; WHO: World Health Organization.

## Competing interests

The authors declare that they have no competing interests.

## Authors’ contributions

DL contributed to the analysis and interpretation of data and drafted the manuscript. GP and EJ contributed to the interpretation of data and revised the manuscript. AA contributed to the conception of the study. ALV and AF contributed to revision of the manuscript. GR designed and supervised the study. All authors read and approved the final manuscript.

## Authors’ information

DL, CépiDc - Inserm, AP-HP, Internship; GP, CépiDc - Inserm, Ph., Computer scientist; EJ, CépiDc - Inserm, Mr., Research Engineer; AA, CépiDc - Inserm, MD, PhD, Coding expert; ALV, CépiDc - Inserm, MD, Coding department director; AF, Institut de veille sanitaire, PhD, Coordination department of alerts and regions; GR, CépiDc - Inserm, PhD, Director

## Supplementary Material

Additional file 1 **Iris software.** Short description of Iris Software. Click here for file

Additional file 2 **Rules for mortality coding.** Definition of rules for mortality coding from ICD-10. Click here for file
