# Supplementary material for: Quality comparison of electronic versus paper death certificates in France, 2010
Source: Popul Health Metr. 2014 Feb 17;12:3. doi: 10.1186/1478-7954-12-3 (PMC3931487; doi:10.1186/1478-7954-12-3)
Supplement: Additional file 1 — Iris software. Short description of Iris Software. [file 1478-7954-12-3-S1.docx]

## Addition file 1 Iris software

Iris is an interactive coding system dedicated to the coding of causes of death and to the selection of the underlying causes of death. The aim of Iris is twofold.

- To propose a language independent system. Language aspects are separated from the software and stored into tables in a database.
- To improve international comparability. Iris is based on the international form of death certificate provided by WHO. The causes of death coding follow ICD10 rules and guidelines. On the other hand, the selection of the underlying cause of death is entirely based on components of the MMDS software developed by the US National Centre for Health Statistics (NCHS). Because Iris includes these components, it is in phase with the updates of the MMDS for the selection of the underlying cause of death.

Iris has been designed and developed on the basis of a collaboration of several countries including France, Germany, Hungary, Italy, Spain, Sweden and United States.

Given the objectives of the Iris project, it is important to keep Iris an international product. Iris is a free software but not open source. Iris evolutions are decided by a user group opened to each country using Iris.
