# Supplementary material for: Quality comparison of electronic versus paper death certificates in France, 2010
Source: Popul Health Metr. 2014 Feb 17;12:3. doi: 10.1186/1478-7954-12-3 (PMC3931487; doi:10.1186/1478-7954-12-3)
Supplement: Additional file 2 — Rules for mortality coding. Definition of rules for mortality coding from ICD-10. [file 1478-7954-12-3-S2.docx]

## Addition file 2 Rules for mortality coding

| **Rules** | **Definition according to the International Statistical Classification of Diseases and Related Health Problems** |
| --- | --- |
| **General Principle** | The General Principle states that when more than one condition is entered on the certificate, the condition entered alone on the lowest used line of Part I should be selected only if it could have given rise to all the conditions entered above it. |
| **Selection Rule 1** | If the General Principle does not apply and there is a reported sequence terminating in the condition first entered on the certificate, select the originating cause of this sequence. If there is more than one sequence terminating in the condition mentioned first, select the originating cause of the first-mentioned sequence. |
| **Selection Rule 2** | If there is no reported sequence terminating in the condition first entered on the certificate, select this first-mentioned condition. |
